# Investigation of Copper Cysteamine Nanoparticles as a New Type of Radiosensitiers for Colorectal Carcinoma Treatment

**DOI:** 10.1038/s41598-017-09375-y

**Published:** 2017-08-24

**Authors:** Zhipeng Liu, Li Xiong, Guoqing Ouyang, Lun Ma, Sunil Sahi, Kunpeng Wang, Liangwu Lin, He Huang, Xiongying Miao, Wei Chen, Yu Wen

**Affiliations:** 1 Department of General Surgery, Second Xiangya Hospital, Central South University, Changsha, Hunan 410011 PR China; 2 Department of Physics and the SAVANT Center, The University of Texas at Arlington, Arlington, Texas 76019-0059 USA; 3 State Key Laboratory for Powder Metallurgy, Central South University, Changsha Hunan, 410083 PR China; 4 Department of Histology and Embryology, Xiangya School of Medicine, Central South University, Changsha, Hunan 410078 PR China

## Abstract

Copper Cysteamine (Cu-Cy) is a new photosensitizer and a novel radiosensitizer that can be activated by light, X-ray and microwave to produce singlet oxygen for cancer treatment. However, the killing mechanism of Cu-Cy nanoparticles on cancer cells is not clear yet and Cu-Cy nanoparticles as novel radiosensitizers have never been tested on colorectal cancers. Here, for the first time, we investigate the treatment efficiency of Cu-Cy nanoparticles on SW620 colorectal cells and elucidate the underlying mechanisms of the effects. The results show that X-ray activated Cu-Cy nanoparticles may kill SW620 cancerscells is in a dose-dependent manner. The JC-1 staining shows the mitochondrial membrane potential is decreased after the treatment. The observations confirm that Cu–Cy nanoparticles may improve X-ray radiotherapy on cancer treatment and X-ray activated Cu-Cy nanoparticles can be efficiently destroy colorectal cancer cells by inducing apoptosis as well as autophagy. As a new type of radiosensitizers and photosensitizers, Cu-Cy nanoparticles have a good potential for colorectal cancer treatment and the discovery of autophagy induced by X-ray irradiated Cu-Cy nanoparticles sheds a good insight to the mechanism of Cu-Cy for cancer treatment as a new radiosensitizers.

## Introduction

Cancer is a very complicated disease and more 1,685,210 new cancer cases were expected to be diagnosed in 2016. About 595,690 Americans died of cancer in 2016, which translates to about 1,630 people per day^1^. Cancer is the second most common cause of death in the US, exceeded only by heart disease, and accounts for nearly 1 of every 4 deaths. Colorectal cancer (CRC) is the third most common cancer and threatens the life and health of humans worldwide, with more than 1.2 million new cases and 600,000 deaths per year^2^. At present, surgical resection is the standard treatment for CRC. The patients’ 5-year relative survival rate is approximately 65%. The relative survival rate of patients with unresectable metastatic lesions decreases to only 5%^3^. Early diagnosis and effective treatment are the hope for cancer patients. Treatments include surgery, radiation, chemotherapy, hormone therapy, immune therapy, and targeted therapy. Even though these treatments are generally good, they have some shortcomings in one way or another. For examples, radiotherapy is one of the most common and most effective methods on cancers but it has a high side-effect at high doses^4–6^. More than 50% of cancer patients undergo radiotherapy at least once during their treatment^7^. One of the greatest challenges in radiotherapy is that ionizing radiation affects both the healthy tissue and the cancerous tissue. Since the tissue around tumor is affected by radiation, there is a limitation in increasing radiation dose. Therefore, it would be significantly important to improve the radiation efficacy and reduce its doses or to have a method that could enhance the dose to the tumor tissue while prevent the radiation to the surrounding tissue. Many methods have been explored to reduce the side-effects of radiation therapy, such as the combination of radiotherapy with photodynamic therapy^8–11^ or chemotherapy^12^ and immunotherapy^13^. One effective method to reduce the side-effects and enhance the killing efficacy is to use radiosensitizers to assistant the radiation treatment^14^. Radiosensitizers are adjunctive treatments which make tumor cells more susceptible to radiation. They are designed and made to improve tumor cell killing while having much less effect on normal tissues. Many materials have been reported as radio sensitizers. Recently, nanoparticle radiosensitizers, such as carbon nanotubes^15^ gold nanoparticles^16–18^ and hafnium oxide nanoparticles^19^, have been extensively investigated to as novel radiosensitizers. Here, for the first time, we explore copper cysteamine (Cu-Cy) nanoparticles as a novel radiosenstizers for colorectal carcinoma treatment. Cu-Cy nanoparticles are different from other nanoparticle radiosensitizers because Cu-Cy is also a photosensitizers that can be activated not only by regular light but also by X-ray, microwave and ultrasound to generate reactive oxygen species^10, 20, 21^. Here in this work, we report the phenomena of Cu-Cy NPs for radiation improvement and the mechanisms for cancer cell destruction.

## Results

### X-ray activated Cu-Cy radiosensitizers may inhibit the proliferation of human colorectal cancer cells

**Figure 1:**
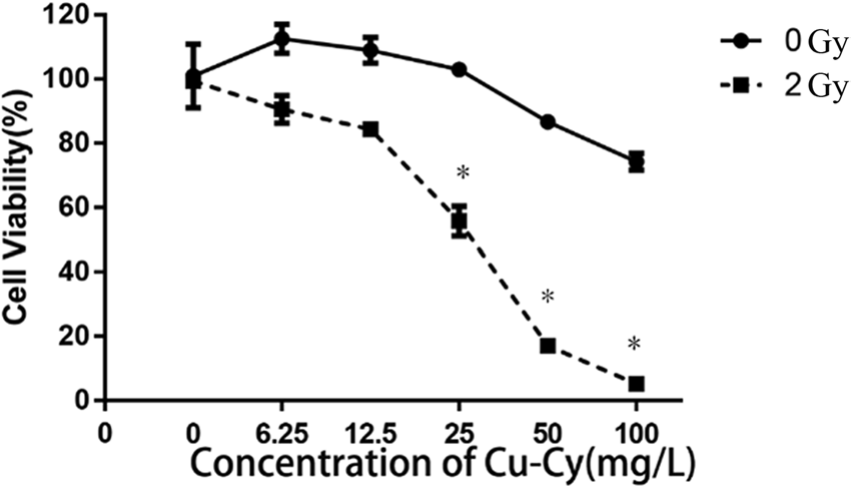
Effects of Cu-Cy nanoparticle radiosensitizers on SW620 cells upon X-ray irradiation (*P < 0.05 compared with control group).

To test the effect of Cu-Cy nanoparticle radiosensitizers on colorectal cancer, cell proliferation was examined by CCK8 assay. As shown in Fig. 1, there is no significant toxicity in treatment with Cu-Cy or X-ray irradiation alone. However, the cell proliferation was severely decreased due to combination of Cu-Cy nanoparticles and X-ray irradiation. X-ray activated Cu-Cy nanoparticles significantly inhibited cell growth in a dose-dependent manner. Cu-Cy doses of 25 and 50 mg/L with X-ray irradiation resulted in cell growth inhibition rates of 44.3 and 82.97% at 24 h, respectively. These results demonstrate that Cu-Cy nanoparticles activated by X-ray could inhibit the proliferation of SW620 human colorectal cancer cells. This also indicates that Cu-Cy nanoparticles can improve X-ray radiotherapy for colorectal cancer treatment as a new type of radiosensitizers.

We also investigated the dose dependence of the cell killing for different concentration as shown in Figure S1 for different concentrations and different X-ray doses. It shows that the efficacy is dependent on both the particle concentration and the X-ray dose. At a low dose of around 1 Gy, there is almost no effects. When the X-ray dose is 2 or 3 Gy, the killing effect is largely increased. As compared to the X-ray treatment alone at the dose of 2 or 3 Gy, the cell killing is increased about 40–60% with Cu-Cy nanoparticles. Interestingly, at the dose of 4 Gy, the killing effect is not increased much using Cu-Cy nanoparticles and the overall effect for the 4 Gy is even lower than that at 3 Gy. This is due to the fact that the X-rays can activate Cu-Cy nanoparticles to produce reactive oxygen species to kill cancer cells^10^. Therefore, the efficacy is determined by the radiation effects and the ROS effects, and it is reasonable to understand that the killing efficacy is not simply increased with the radiation dose. This is actually a very good phenomenon as the optimized dose may result the best outcome.

### Cell Uptake and Morphology Upon PDT Treatment

**Figure 2:**
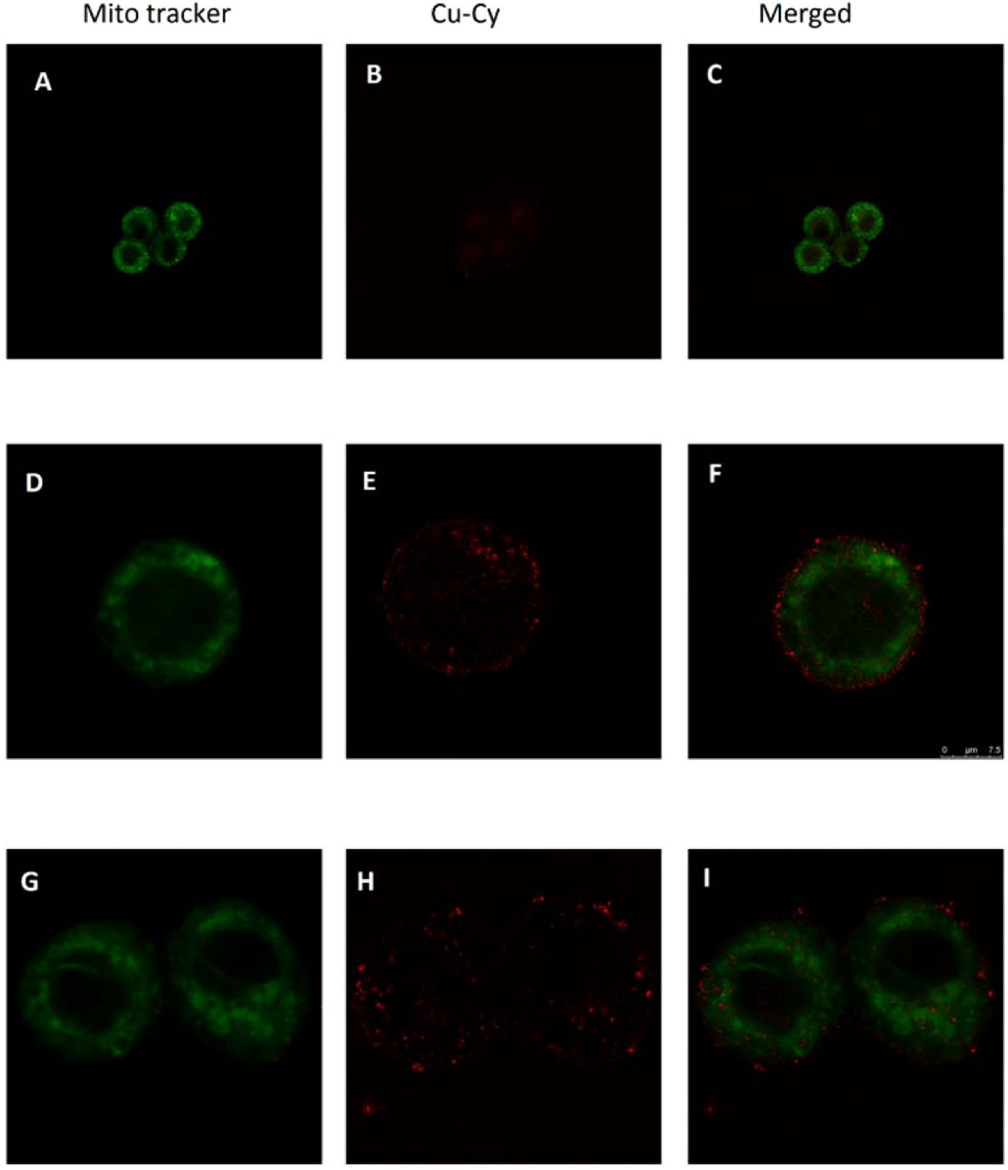
Intracellular distribution of Cu-Cy with CLSM in SW620 (**A**–**I**). The green fluorescence representing the locations of MitoTracker Green probes which are identical to those of the Mitochondria in SW620 cells (**A**,**D**,**G**), the auto-fluorescence of Cu-Cy (**B**,**E**,**H**) and the overlapped fluorescence of the Mitochondria tracker and Cu-Cy (**C**,**F**,**I**). Scale bars are 7.5 μm.

Cell uptake is important for both imaging and treatment. Shown in Fig. 2 is the uptake after 4 hours incubation. The green is the imaging of the mitochondria by a MitoTracker Green probe. The red imaging is from Cu-Cy nanoparticles. The images show that after 4 hours, Cu-Cy nanoparticles got into the cells and some nanoparticles are surrounding the mitochondria. This is very interesting because mitochondria is the target for ROS based therapy^22^.

**Figure 3:**
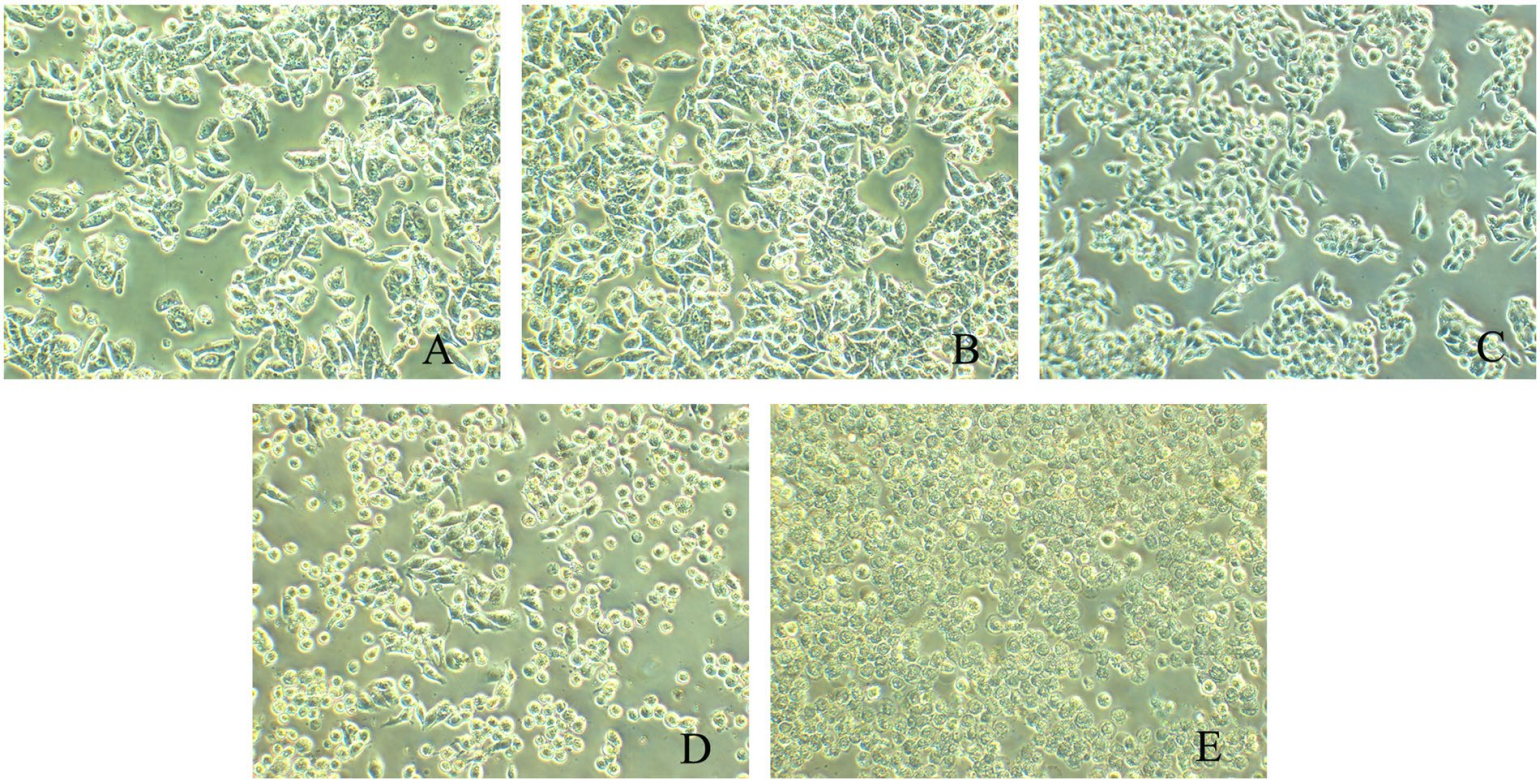
Changes in cell morphology after treatment with X-ray alone (**B**) and Cu-Cy nanoparticles alone 50 mg/L (**C**); the combination of X-ray and Cu-Cy nanoparticles at the concentrations of 50 mg/L (**E**) and 100 mg/L (**F**). A is the control. The X-ray dose is 2 Gy for each treatment.

The change of the cell morphology may give some insights about the interaction of X-ray activated Cu-Cy nanoparticles on cells. As shown in Fig. 3, under the X-ray irradiation at 2 Gy, there is no changes observed on the cells, the SW620 cells are long semispherical shape and the cells grew in good conditions as in the control. For the cells treated with Cu-Cy nanoparticles alone, almost no changes are observed and the cells grew in good conditions as in the control. This indicates Cu-Cy nanoparticles have no or very low toxicity. However, upon the treatment by X-ray and Cu-Cy together, the cells became spherical in shape, they shrink and lost their light reflection and the separations in between the cells are increased. Obviously, the cells were damaged and defects were seen among the cells. Along with the damage, some cells were dead, and as the Cu-Cy concentration is increased, more and more cells suffered from apoptosis or/and necrosis.

### X-ray activated Cu-Cy nanoparticles induce apoptotic cell death

**Figure 4:**
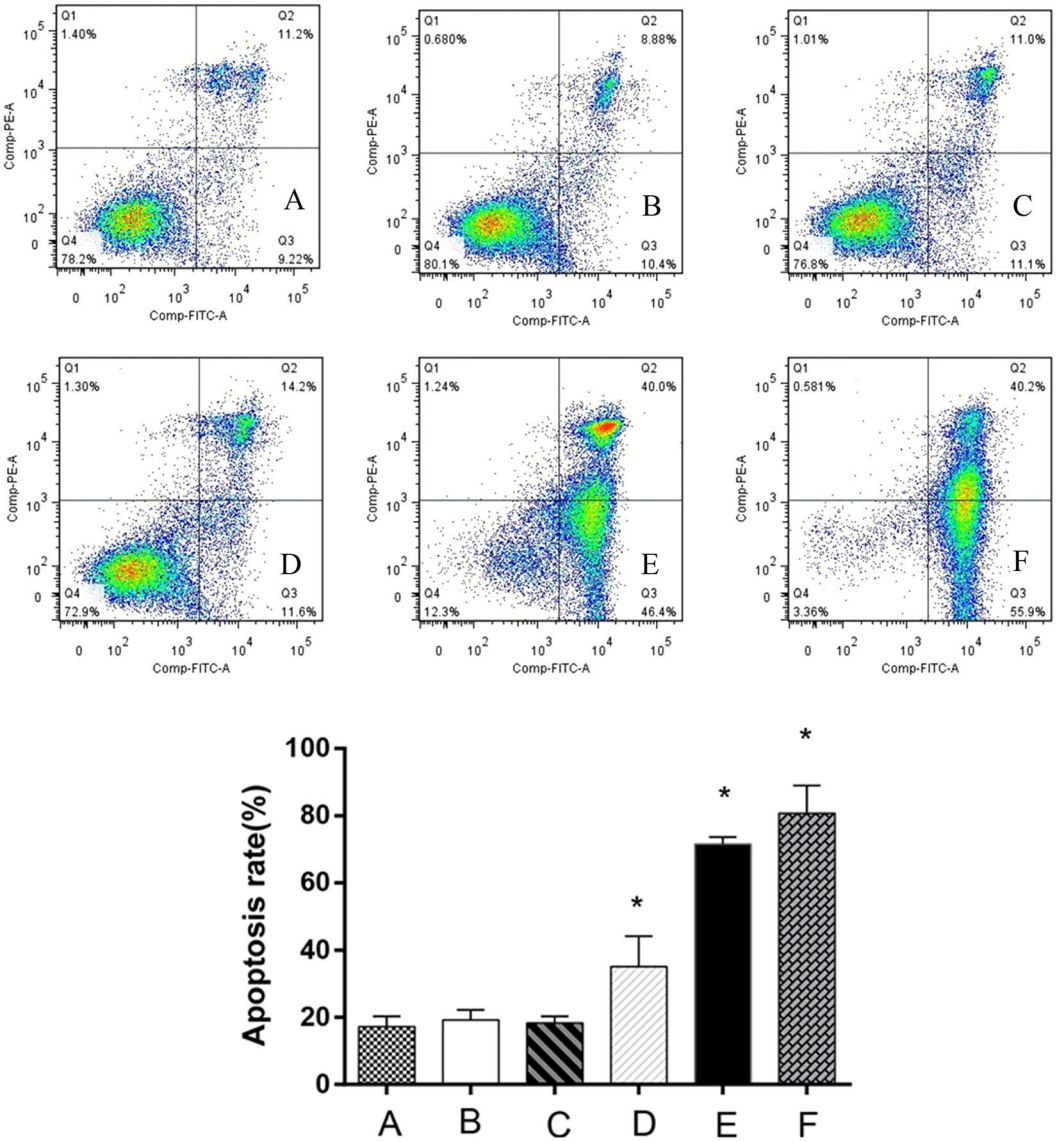
Analysis of mitochondrial membrane potential in SW620 cells in different groups by flow cytometry. (**A**) Normal control group; (**B**) X-ray irradiation group; (**C**) Cu-Cy group (50 mg/L) (**D**) Cu-Cy-PDT (25 mg/L); (**E**) Cu-Cy-PDT (50 mg/L); (**F**) Cu-Cy-PDT (100 mg/L) *P < 0.05 compared with normal control group.

To assess whether the growth inhibitory effects of SW620 were associated with the induction of apoptosis, cell death was quantified by Annexin V-FITC/PI staining. The rates were 17.20 ± 3.09%, 19.21 ± 2.95%, and 18.24 ± 2.05% for the normal control group, Cu-Cy group, and irradiation-alone groups, respectively (Fig. 4). Cu-Cy doses of 25 mg/L, 50 mg/L and 100 mg/L with X-ray irradiation remarkably induced 35.09 ± 9.08%, 71.53 ± 1.22%, 80.74 ± 4.74% cell apoptosis in SW620 cells. The apoptosis rates in both Cu-Cy-PDT groups (what groups?) were significantly higher than those in the control. These observations indicate that X-ray activated Cu-Cy nanoparticles are highly associated with apoptosis.

### X-ray activated Cu-Cy nanoparticles may reduce mitochondrial membrane potential (MMP)

**Figure 5:**
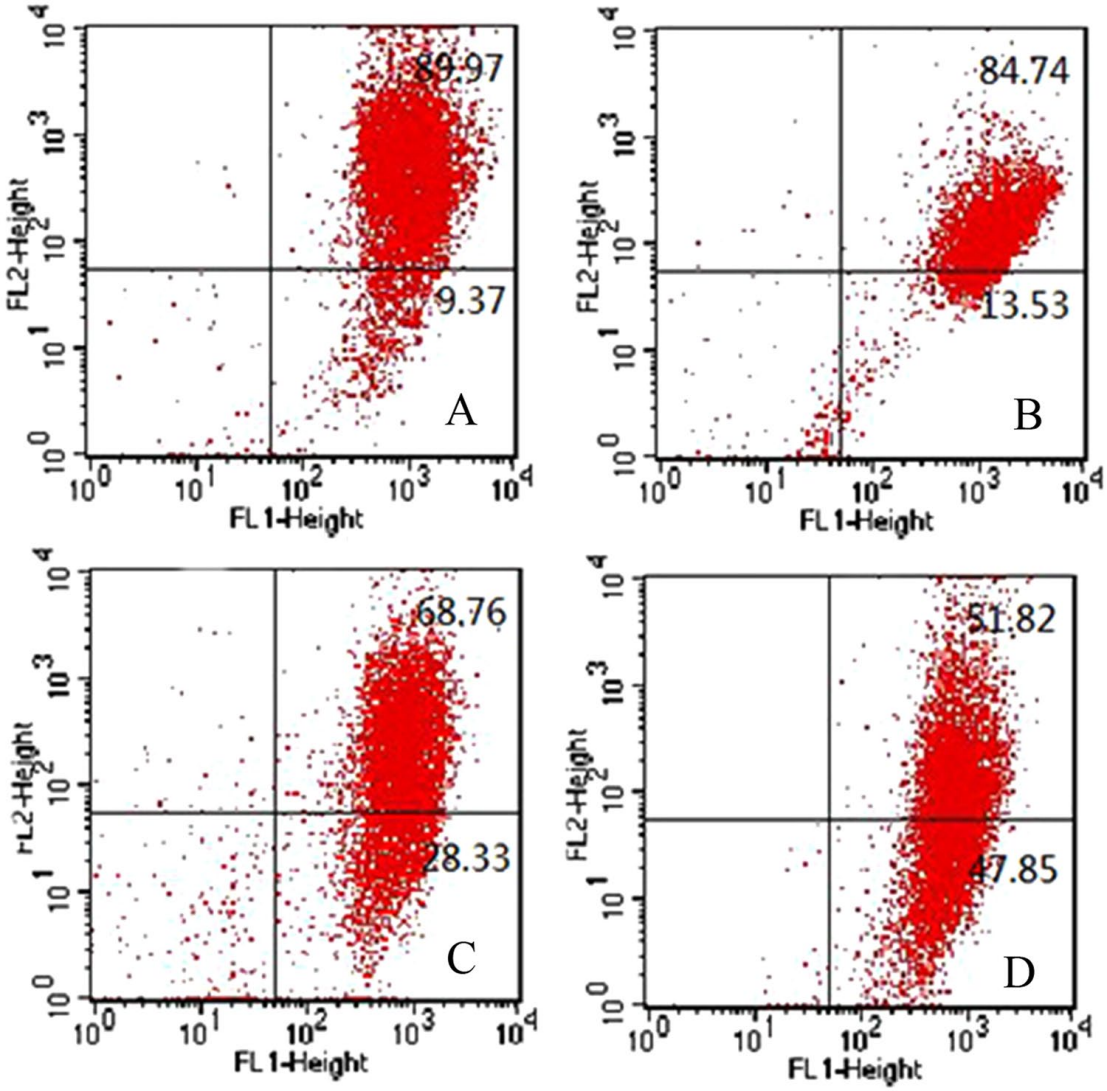
The plot of JC-1 red verse green emission from flow cytometry on mitochondrial membrane potential. (**A**) Normal control group; (**B**) X-ray irradiation group; (**C**) Cu-Cy-PDT (50 mg/L); (**D**) Cu-Cy-PDT (100 mg/L).

MMP is an important parameter of mitochondrial function used as an indicator for cell death. The collapse of the mitochondrial membrane potential coincides with the opening of the mitochondrial permeability transition pores, resulting in the release of cytochrome c into the cytosol, which in turn downstream events in the apoptotic cascade. The dissipation of the mitochondrial inner membrane potential is considered as an early sign of apoptosis. Thus, the examine of the status of the MMP may typify the mode of the X-ray activated Cu-Cy nanopaticles for cell death. The MMP was measured by JC-1 staining. The mitochondrial-specific dual-fluorescence probe (JC-1) exhibits a potential-dependent accumulation in mitochondria. In healthy cells, the mitochondria are polarized and JC-1 accumulates in the mitochondria as aggregates with a red emission at 590 nm. In cells with decreased MMP, the mitochondria are depolarized to block the transmission of JC-1 into the mitochondria, JC-1 remains in the cytosol as monomeric form with a green emission at 535 nm. So, the emission color of JC-1 is a simple and good probe for MMP. As shown in Fig. 5, for the cells treated by Cu-Cy or X-ray-alone, respectively, the red emission from JC-1 aggregates is dominated, indicating that the MMP is preserved. However, for the cells treated with X-ray and Cu-Cy nanoparticles, the emission of JC-1 is shifted from red to green, indicating the decrease of MMP and the depolarization of the mitochondria, the early stage of cell apoptosis. These observations again prove that Cu-Cy nanoparticles mediated X-ray are highly associated with apoptosis.

### Expression of Bax, Bcl-2, LC3B and P62 in cells treated by X-ray and Cu-Cy nanoparticles

**Figure 6:**
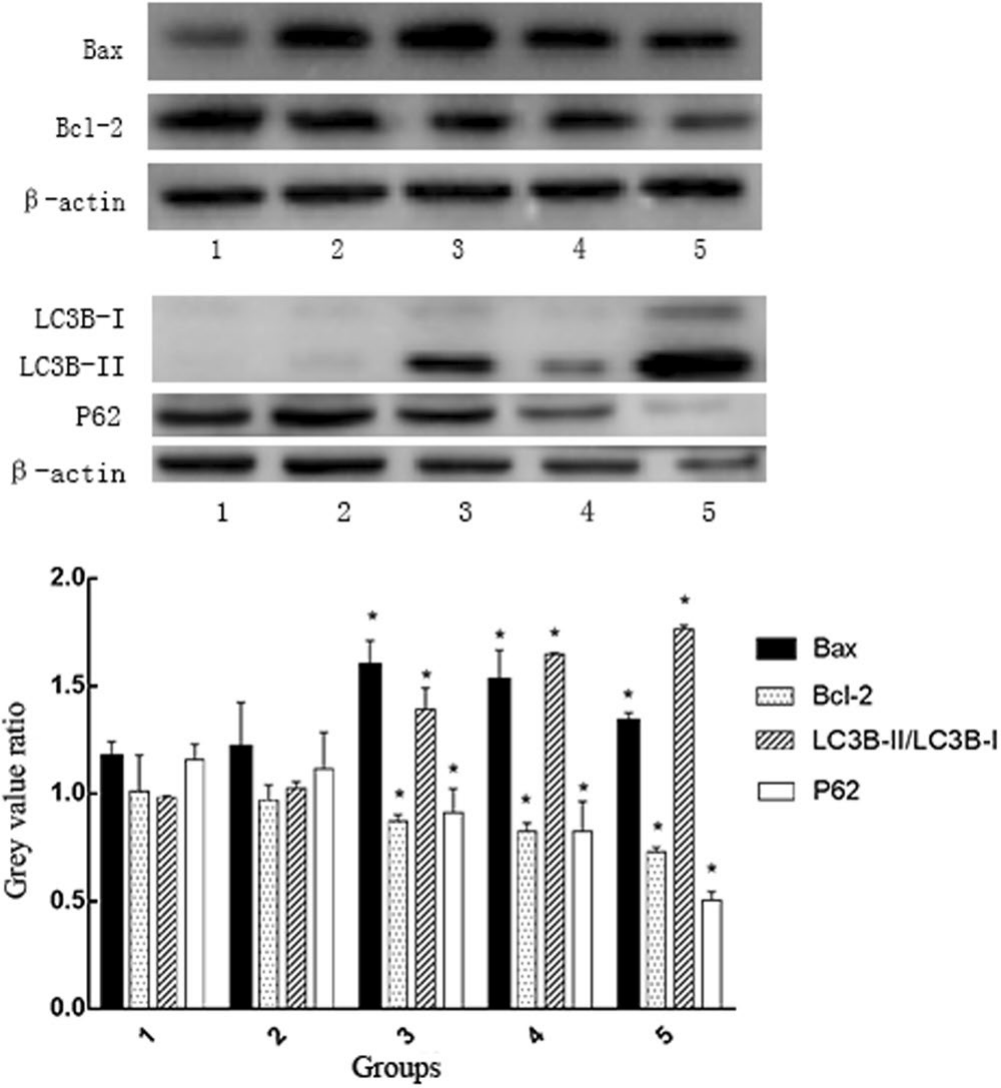
The protein expression levels of Bax, Bcl-2, LC3B, and P62 was detected by western blot analysis. SW620 cells were seeded into a 6-well plate at a density of 5 × 10^5^ cells per well, cultured overnight and treated with or without Cu-Cy-PDT. The cells were lysed for 30 minutes in 1 × RIPA buffer that contained protease and phosphatase inhibitors. Samples containing equal amounts of protein (25 μg) were resolved on SDS-PAGE in a 10–15% gel and then transferred to a polyvinylidene fluoride (PVDF) membrane (Millipore, Billerica, MA, USA). The membrane was then blocked in 5% non-fat milk for 2 h at room temperature, incubated with primary antibody Bax, Bcl-2, ATG7, LC3B, and Actin antibody (Cell Signaling, Danvers, MA, USA) overnight at 4 °C, washed with TBST, and incubated with a secondary antibody for 2 h at room temperature. The immunoblots of the incubated membranes were visualized with an enhanced chemiluminescence (ECL) Kit (CW Bio).

In order to understand better the mechanisms for X-ray induced Cu-Cy nanoparticle effects on the cell growth and apoptosis, we then examined the expression levels of the apoptotic and autophagy-related proteins Bax, Bcl-2, P62, and LC3B. Western blotting shows that X-ray activated Cu-Cy nanoparticles may cause an increase in the expression of Bax in a dose-dependent manner, whereas the expression of Bcl-2 was reduced in SW620 cells. Figure 6 clearly shows that SW620 cells treated with X-ray activated Cu-Cy nanoparticles have a notable decrease in p62 expression levels compared with blank control at 24 hours after the treatment. SW620 cells had a dose-dependent decrease in p62 expression. We next tracked the conversion of LC3B-I to LC3B-II, which is a marker for autophagic vesicles and autophagic activity. When autophagy is induced, LC3B-I is conjugated to phosphatidylethanolamine (PE) to form LC3B-II. Therefore, the conversion of LC3B-I to LC3B-II is correlated with the autophagic reflux. In Fig. 5, the conversion of LC3B-I to LC3B-II was dramatically increased in SW620 cells at 20 mg/L for 24 h after the PDT, and this proportion became more evident at 50 mg/L after PDT. All these indicate that X-ray activated Cu-Cy nanoparticles induced autography on the SW620 cells.

1: Normal control group; 2: X-ray irradiation group; 3: Cu-Cy-PDT (50 mg/L); 4: Cu-Cy-PDT (50 mg/L); 5: Cu-Cy-PDT (100 mg/L) *P < 0.05 compared with control group; the X-ray irradiation group.

### Observation of autophagic vacuoles induced by X-ray activated Cu-Cy nanoparticles

**Figure 7:**
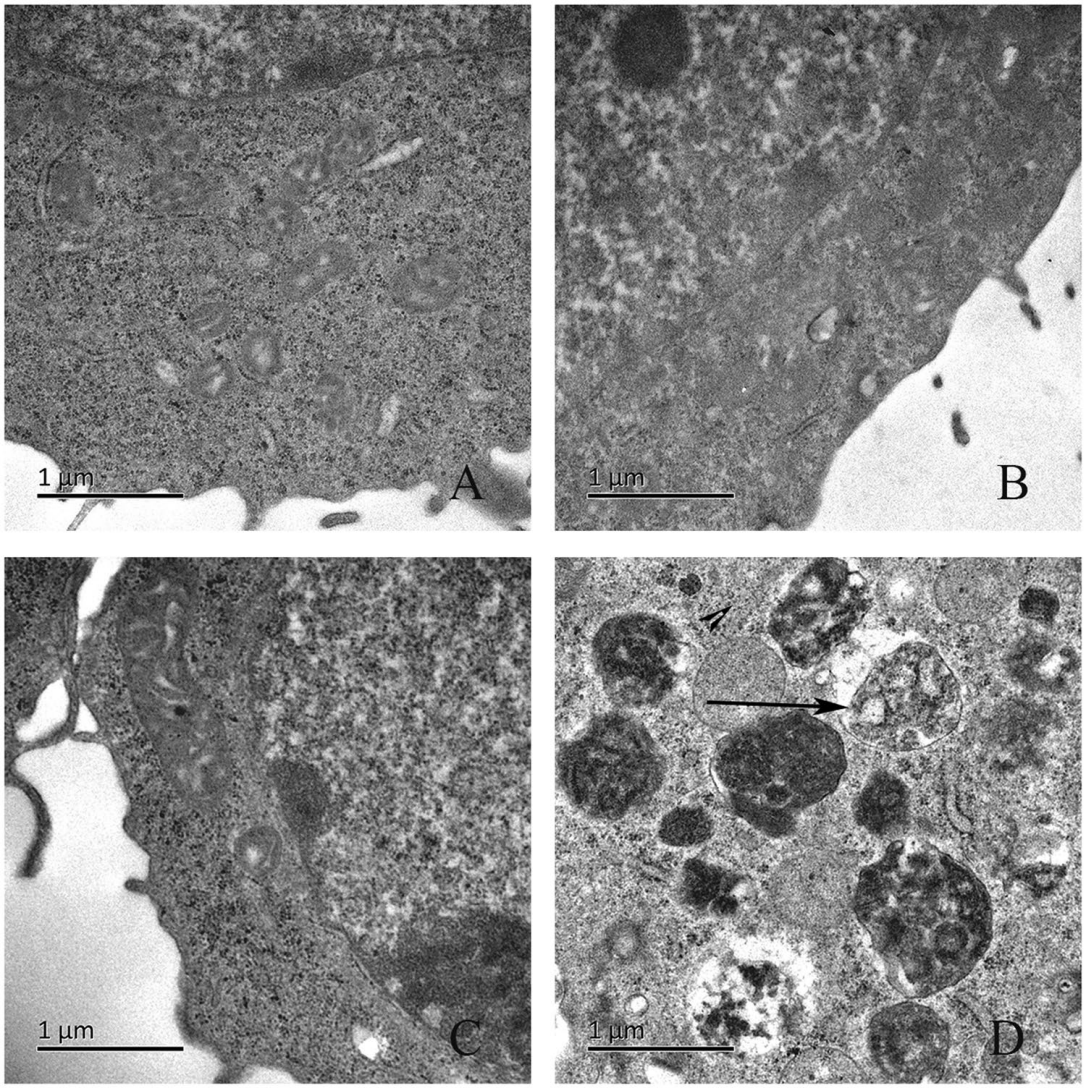
Changes of SW620 colorectal cancer cells under transmission electron microscope after PDT. (**A**) Normal control group (×20000); (**B**) X-ray irradiation group (×20000); (**C**) Cu-Cy (50 mg/L) (×20000); (**D**) Cu-Cy-PDT (50 mg/L) (×20000). The arrow indicates the typical appearance of autophagosomes.

In order to prove that X-ray activated Cu-Cy nanoparticles can induce autophagy in SW620 cells, the treated cells were morphologically examined by TEM, which is considered the “gold standard” for autophagy^23^. The results show that the control cells have normal morphologies for their cytoplasm and organelles (Fig. 7A). For the cells treated with X-ray (2 Gray) or Cu-Cy alone, almost no damages or vacuoles were observed (Fig. 7B and C). However, a high level of double membrane-bound vacuoles marked by the arrowheads were observed inside SW620 cells after 1 h treatment by X-ray and Cu-Cy nanoparticles. The vacuoles were found with engulfed bulk cytoplasm and cytoplasmic organelles, which are typical evidences for autophagosomes (Fig. 7C). These observations support that X-ray irradiated Cu-Cy nanoparticles induce autophagy in SW620 cells.

## Discussion

For radiation treatment or radiotherapy, the dose is a critical issue because it will not damage only the cancer cells but also the healthy cells surrounding the cancer cells if the dose is too high. To improve the efficacy and reduce the side effects, here, we report that Cu-Cy nanoparticles can improve radiation treatment as a new radiosensitizer. Also, Cu-Cy is an effective photosensitizer that can be activated by UV light, visible light, X-rays and microwaves^10, 20, 21^. We have two strong pieces of evidence to demonstrate that Cu-Cy is also a new and efficient photosensitizer. The first is that the singlet oxygen production efficiency excited by UV at 365 nm in Cu-Cy nanoparticles is almost the same as in protoporphyrin IX (PPIX) which is a well-known, commercially available photosensitizer^24, 25^ (see Supplementary Figure S2 left). In our pilot studies, we used the p-nitrosodimethylaniline (RNO)-imidazole(ID) method to detect singlet oxygen^26^. RNO-ID is a very sensitive method for singlet oxygen detection, however, this method is not very specific for singlet oxygen as it may be reacted with other reactive oxygen species too. To further prove the Cu-Cy produce singlet oxygen, we apply NaN_3_, a selective singlet oxygen quencher to probe singlet oxygen^27^. As shown in the Supplementary Figure S2 -center, when NaN_3_ was added to the Cu-Cy solution, the production of ROS from RNO-ID measurement is largely reduced because of the quenching of singlet oxygen by NaN_3_. This strongly supports that the ROS produced in Cu-Cy is mainly singlet oxygen.

The second evidence is that the Cu-Cy decay lifetimes are very long as reported in our recent paper^21^. The luminescence of Cu-Cy nanoparticles has two sufficiently long decays – one being 7.399 microseconds and the slower one at 0.363 milliseconds which are in the same range of luminescence decay lifetimes from triplet states of photosensitizers^24, 26^. These indicate that Cu-Cy has a triplet state which is very important for photosensitizers.

**Figure 8:**
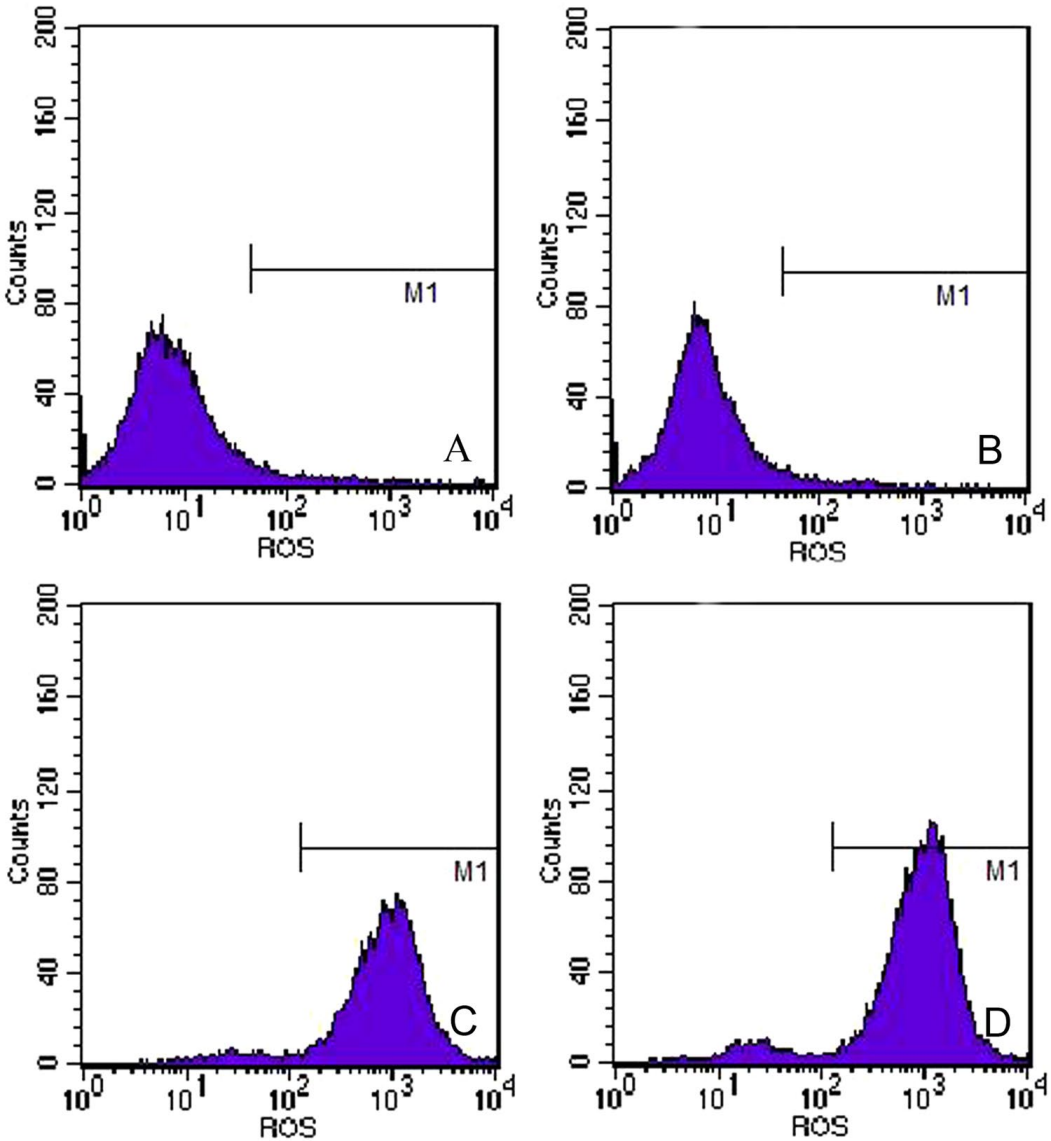
Singlet oxygen production in SW620 cells measured with HDFH-DA. The X-ray irradiation dose is 2 Gy.

As shown in the Supplementary Figure S2-right, upon X-ray irradiation, the singlet oxygen produced in Cu-Cy is much higher than in PPIX. This unique characteristic makes Cu-Cy capable for deep cancer treatment. The efficacy of X-ray activated Cu-Cy nanoparticles on colorectal cancer cells is also attributed to production of singlet oxygen and other reactive oxygen species^10^. The ROS production on SW620 cells was measured with singlet oxygen sensors (DCFH-DA) and the results are shown in Fig. 8. For the control and the cells treated with X-ray only, the ROS production is very low, while for the cells treated with X-ray activated Cu-Cy nanoparticles, the ROS production is increased 1000 times and the ROS amount is higher for higher concentrations of Cu-Cy. The ROS production is coincident with the treatment efficacy - both are in dose-dependent manners. This further proves that the cancer cell killing efficacy is attributed to the production of ROS and singlet oxygen generated from Cu-Cy nanoparticle photosensitizers upon X-ray irradiation.

X-ray activated Cu-Cy nanoparticles significantly inhibited the proliferation of human colorectal cells in a dose-dependent manner. Apoptosis has been reported as the predominant cell death pathway for ROS mediated tumor inhibition in several cancer cell lines^28^. In this study, it is observed that X-ray activated Cu-Cy nanoparticles significantly induced apoptotic cell death, as evidenced by the increased ratio of Bax ⁄ Bcl-2 proteins in cells treated with X-ray and Cu-Cy nanoparticles.

Autophagy is a homeostatic cellular recycling mechanism and has recently attracted the interest in the field of cancer research due to its designation as programmed cell death type II^29^. 33 Although there are a few reports of ROS induced autophagy, the induction of autophagy by singlet oxygen is still not well understood^22, 30^. 34 Our results show that X-ray activated Cu-Cy nanoparticles dramatically increased the levels of the autophagy-specific proteins LC3-II and P62 and induced the accumulation of autophagic vacuoles detected by transmission electron microscopy. These observations indicates that Cu-Cy nanoparticle mediated radiotherapy induces not only apoptosis but also autophagy in SW62 cells.

Many therapeutic treatments have been shown to modulate autophagy signaling, although it is still unclear whether autophagy represents a survival or a suicide mechanism in treated cells. Autophagy can ultimately prevent genome damage by clearing away damaged proteins and organelles that drives tumorigenesis. In contrast, autophagy enables tumor cells to tolerate stress and can prolong their survival. When cells attempt to recycle damaged constituents beyond their capacity for recovery, autophagy can become a cell-death pathway^23^. Silencing of the autophagy gene designated Atg7 results in the photosensitization of mouse leukemia L1210 cells to photodynamic effects^31^. 35 However, in human breast cancer MCF-7 cells, silencing of Atg7 may make the cells more resistant to radiation treatment. Collectively, these studies suggest that radiation very likely induces autophagy and apoptosis and that autophagy might play different roles in different cell types and with different photosensitizers^32^. Cu-Cy mediated radiotherapy can lead to both autophagy and apoptosis, which indicates that Cu-Cy as a new photosensitizer not only can be used for deep cancer treatment but also have some special properties that deserve further investigations.

The possibility of using X-ray for photosensitizers (PSs) activation has been reported in literature; however, the results are ambiguous and most studies found that the efficiency of PSs for radiation enhancement is very low^33–36^. In some cases, PSs even decrease the efficiency of accompanying radiation treatments^37^. One possible reason is that PSs like porphyrin have very low stopping power and cannot effectively absorb X-rays for activation. Taken together, this study suggests that Cu-Cy nanoparticle radiosensitizers can be a potential anti-tumor agents for human colorectal cancer.

To summarize, for the first time, the effects of Cu-Cy nanoparticle radiosensitizers are studied on SW620 colorectal cells on radiotherapy improvement. The observations confirm that Cu–Cy nanoparticle radiosensitizers can efficiently improve X-ray radiation to destroy colorectal cancer cells by inducing apoptosis as well as autophagy. As a new type of radiosensitizers, Cu-Cy nanoparticles have a good potential for colorectal cancer treatment. The discovery of autophagy induced by X-ray activated Cu-Cy nanoparticles brings a good insight to the mechanism of Cu-Cy for cancer treatment as a new photosensitizer as well as a novel radiosensitizer.

## Materials and Methods

### Synthesis of Cu–Cy Particles

CuCl_2_·2H_2_O (0.460 g, 2.698 mmol) was dissolved in DI water followed by the addition of cysteamine (0.636 g, 8.244 mmol). After adjusting the pH value to 8 by adding a 2.5 M NaOH solution (8 mL), the solution was stirred for approximately 2 h at room temperature and then heated to its boiling temperature for 30 min. The Cu–Cy particles were obtained by centrifuging and washing the crude product with a solution of DI water and ethanol (v/v = 5:4) five times followed by sufficient sonication. Finally, the particles were dried completely in a vacuum oven at room temperature overnight. A stock solution was made in PBS at a concentration of 1 mg/ml. The solution was kept in the dark at −4 °C and diluted in growth medium prior to use.

### X-ray Irradiation

The X-ray system was provided by Faxitron X-Ray Corp. (IL, USA) and was used for all of the tests. X-ray irradiation (90 kV and 5 mA) was conducted at a dose rate of 0.5 Gy/min.

### Photosensitization

Human colon cancer cell line SW620 was purchased from the cell center of the Xiangya School of Medicine of Central South University. The cells were maintained in RPMI-1640 (Hyclone, USA), containing 10% FBS (Gibco, BRL, USA) and 1% penicillin/streptomycin (Gibco) in a humidified atmosphere of 5% CO_2_ at 37 °C. Cells were used for the experiments when in the logarithmic phase of growth. When cells reached approximately 80% confluence, they were pretreated with different concentrations of Cu-Cy in culture medium for 4 hours. Next, the cells were irradiated with 2 Gy X-ray after replacing the Cu-Cy solution with fresh complete culture medium (with 10% FBS).

### Cell Imaging and Uptake Observation

When cells reached approximately 80% confluence, they were pretreated with Cu-Cy(100 mg/L) in RPMI-1640 medium in a humidified atmosphere of 5% CO_2_ at 37 °C for 4 hours. Next, the cells incubated for 30 minutes with 150 nM MitoTracker Green probe (China, Beyotime). Cells were washed with PBS and then immediately observed under CLSM (Germany, Leica). The excitation wavelength for Cu-Cy was 360 nm. The fluorescence excitation and emission wavelengths of the MitoTracker probe are 490 nm and 526 nm, respectively.

### Cell counting Kit-8 assay (CCK8)

Cell viability was measured by a Cell counting Kit-8 assay(Dojindo, Laboratories, Kumamoto, Japan). SW620 cells (2 × 104 cells/well) were seeded into a 96-well plate and cultured overnight. After photosensitization, they were incubated at 37 °C in 5% CO_2_ for 24 h. Following incubation, the medium was removed, 0.1 ml of cell culture medium and 10 μl of the Cell Counting Kit-8 solution were added to each well, and the cells were incubated for an additional 2 h. We measured the absorbance at 450 nm with a microplate reader (Infinite 200 pro, TECAN).

### Apoptosis Analysis

We used the Annexin V-FITC/PI Apoptosis Detection Kit (Roche, Basel, Switzerland) to detect cell apoptosis. SW620 cells were cultured overnight in 12-well plates at a density of 1 × 10^5^ per well. The cells were divided into four groups according to the treatments that they received: the blank control group, X-ray irradiation group, Cu-Cy group, Cu-Cy-PDT group. At 24 hours after PDT treatment, the cells were collected by trypsinization and washed twice in PBS. They were resuspended in 500 μl 1 × binding buffer and stained with 5 μl of Annexin V-FITC plus 5 μl of PI. Cells were incubated for 10 min at room temperature in the dark. Finally, the fluorescence was immediately analyzed using a FACScan flow cytometer (BD Biosciences, CA, USA).

### Analysis of Mitochondrial Membrane Potential (MMP)

Mitochondrial membrane potential was detected using JC-1 fluorescent dye by a flow cytometry. SW620 cells were incubated with various concentrations of Cu-Cy and then incubated with fresh culture medium containing JC-1 dye (2.5 µg/mL) for 20 min at 37 °C in the dark. After X-ray irradiation, approximately 1 × 10^4^ cells were analyzed on a FACScan flow cytometer. The results are shown as a dot plot graph. In each graph, the y-axis corresponds to JC-1 oligomer-associated red fluorescence, the x-axis corresponds to JC-1 monomer-associated fluorescence, and the shift down of fluorescence from red to green indicates the collapse of mitochondrial membrane potential.

### Western blotting

SW620 cells were seeded into a 6-well plate at a density of 5 × 10^5^ cells per well, cultured overnight and treated with or without Cu-Cy-PDT. The cells were lysed for 30 minutes in 1 × RIPA buffer that contained protease and phosphatase inhibitors. Cell lysates were centrifuged at 13,000 g for 30 minutes at 4 °C. The supernatant was collected, and the protein concentration was determined by a BCA protein assay kit (Beyotime Biotechnology, China). Samples containing equal amounts of protein (25 μg) were resolved on SDS-PAGE in a 10–15% gel and then transferred to a polyvinylidene fluoride (PVDF) membrane (Millipore, Billerica, MA, USA). The membrane was then blocked in 5% non-fat milk for 2 h at room temperature, incubated with primary antibody Bax, Bcl-2, ATG7, LC3B, and Actin antibody (Cell Signaling, Danvers, MA, USA) overnight at 4 °C, washed with TBST, and incubated with a secondary antibody for 2 h at room temperature. The immunoblots of the incubated membranes were visualized with an enhanced chemiluminescence (ECL) Kit (CW Bio).

### Transmission electron microscopy

SW620 cells were seeded into 6-well plates at a density of 5 × 10^5^ cells per well and then fixed for transmission electron microscopy (TEM) analysis at 6 h after Cu-Cy-PDT in 2.5% glutaraldehyde. The fixed cells were postfixed with a 1% OsO4 buffer. Next, the samples were dehydrated in graded alcohol and flat embedded in epon resin. Finally, the cell samples were cut into ultrathin sections (100 nm), stained with 3% lead citrate plus uranyl acetate and observed under an electron microscope (Philips CM20).

### Statistical analysis

All experiments were performed at least in triplicate. The results were expressed as the means ± standard deviation (SD). Student’s t test and one-way analysis of variance (ANOVA) were performed to determine the significant difference between the control and experimental groups using SPSS18.0. P values less than 0.05 were considered significant.

## Electronic supplementary material

supporting data
