# Supplementary material for: Investigation of Copper Cysteamine Nanoparticles as a New Type of Radiosensitiers for Colorectal Carcinoma Treatment
Source: Sci Rep. 2017 Aug 24;7:9290. doi: 10.1038/s41598-017-09375-y (PMC5570927; doi:10.1038/s41598-017-09375-y)
Supplement: Supplementary file 1 — supporting data [file 41598_2017_9375_MOESM1_ESM.pdf]

Supporting Information

Investigation of Copper Cysteamine Nanoparticles as a New Type of Radiosensitiers for Colorectal Carcinoma Treatment

Zhipeng Liu[1+], Li Xiong[1+], Guoqing Ouyang[1], Lun Ma,[2] Sunil Sahi,[2] Kunpeng Wang[1], Liangwu Lin[3], He Huang[4], Xiongying Miao[1], Wei Chen[2*], Yu Wen[1*]

[1]Department of General Surgery, Second Xiangya Hospital, Central South University, Changsha, Hunan 410011, PR China

[2]Department of Physics and the SAVANT Center, The University of Texas at Arlington, Arlington, Texas 76019-0059, USA

[3]State Key Laboratory for Powder Metallurgy, Central South University, Changsha Hunan 410083, PR China

[4]Department of Histology and Embryology, Xiangya School of Medicine, Central South University, Changsha, Hunan 410078, PR China

Correspondence to: Wei Chen, e-mail:weichen@uta.edu

Yu Wen, e-mail:1850129046@qq.com

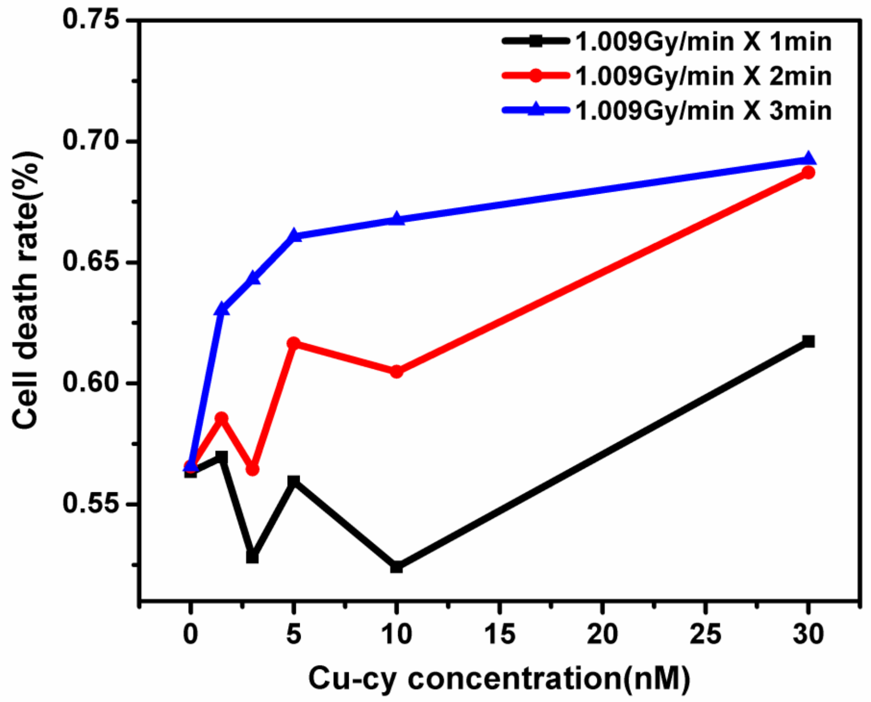

Figure S1. The killing of cancer cells by X-ray induced Cu-Cy with different doses at different concentrations. X-ray operation at 225 kV from a Faxitron X-ray cabinet.

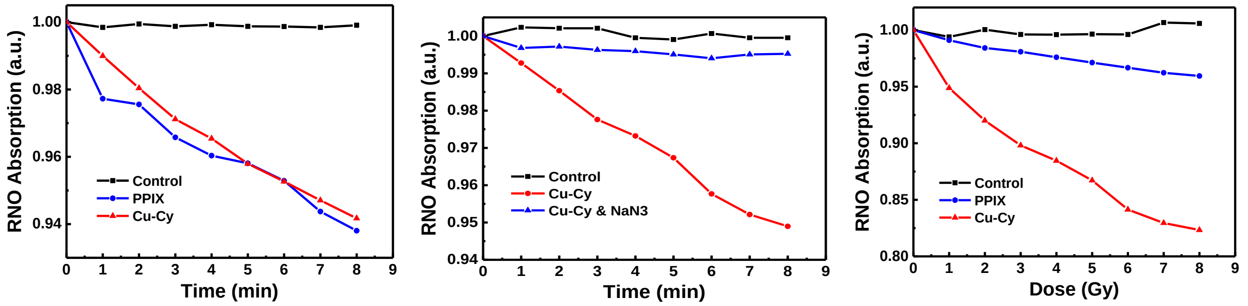

Figure S2. The singlet oxygen produced in Cu-Cy nanoparticles induced by 365 nm light (left) or X-ray (right). When NaN3 is added into Cu-Cy aqueous solution, the RNO signal is quenched largely (center), which indicates the formation of singlet oxygen

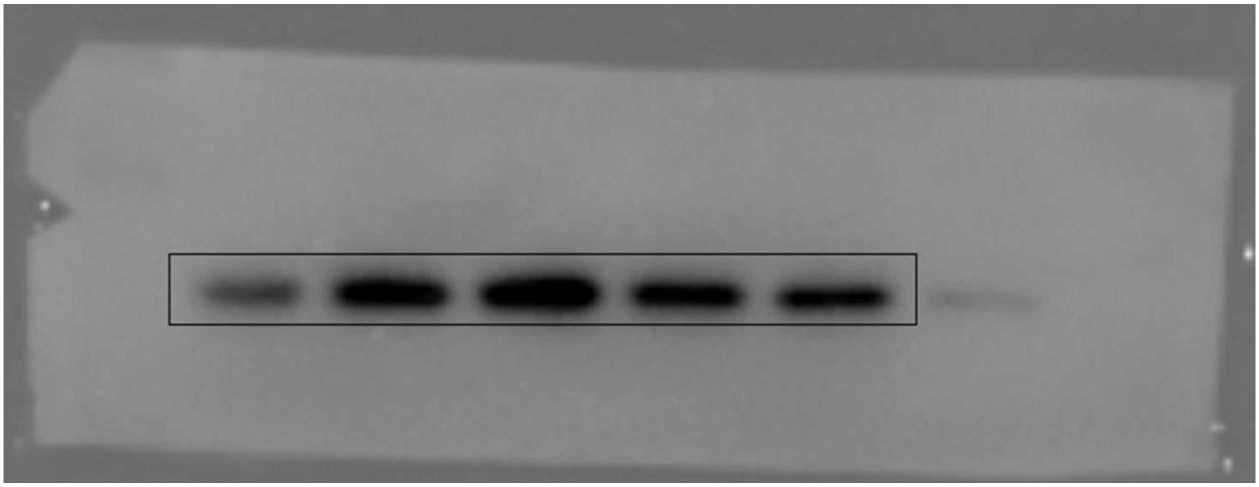

Bax

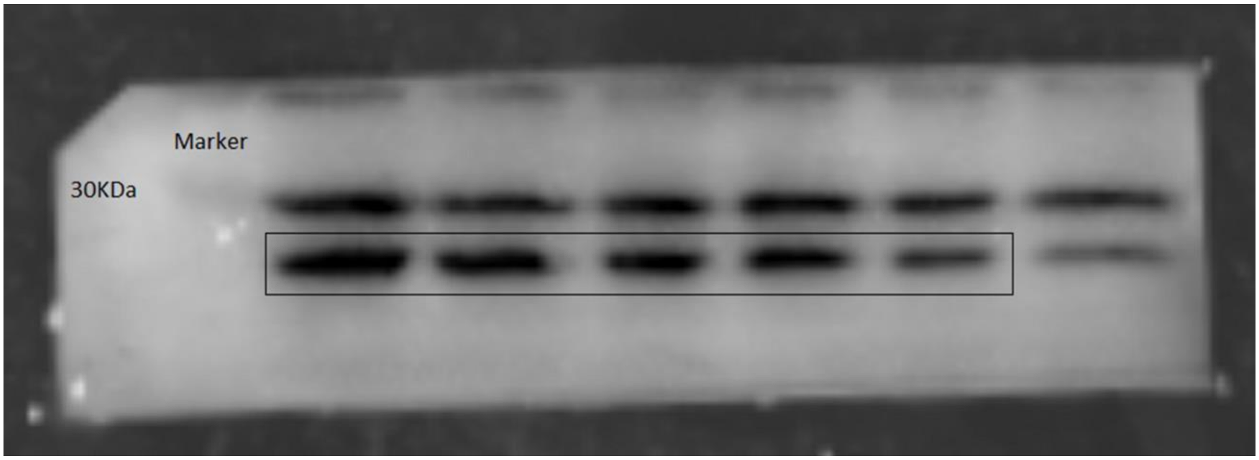

Bcl-2

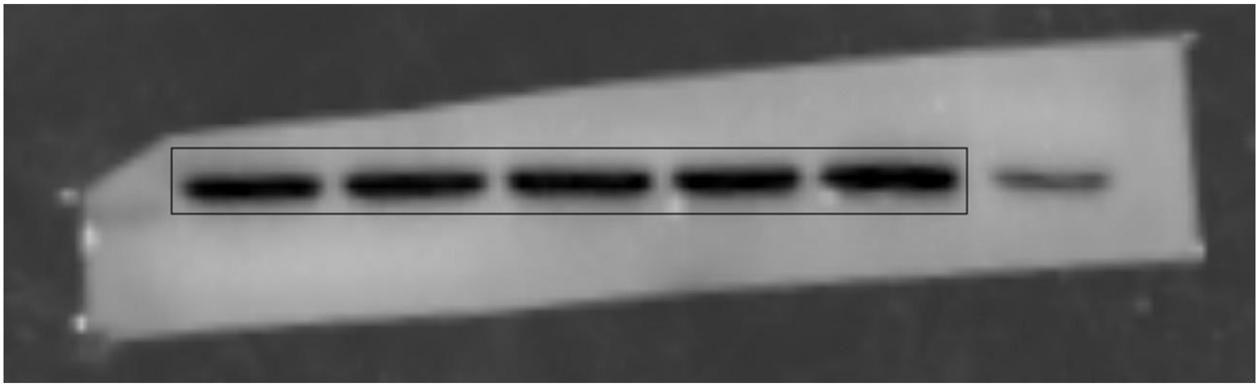

β-actin

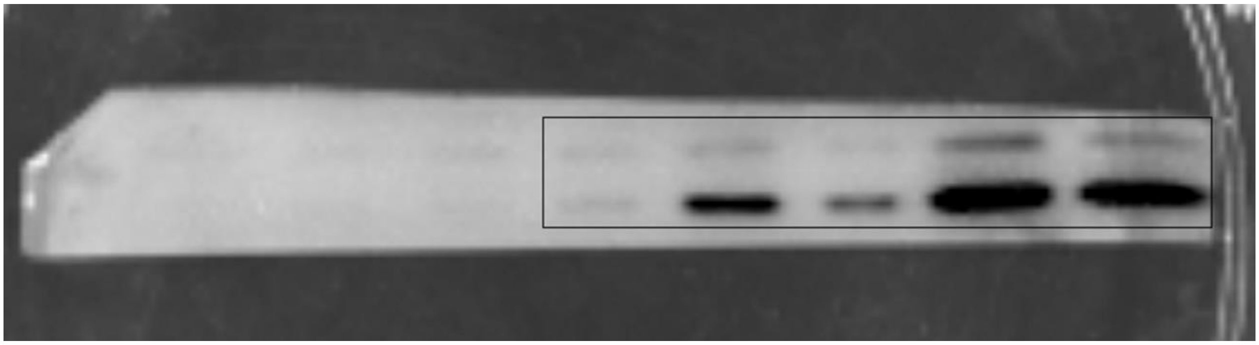

LC3

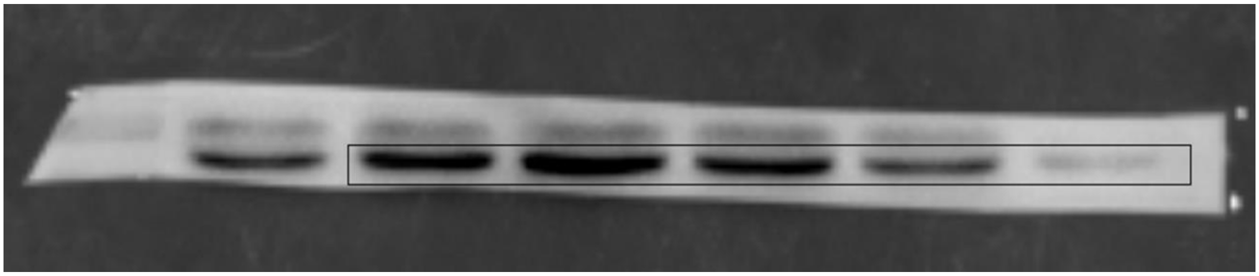

P62

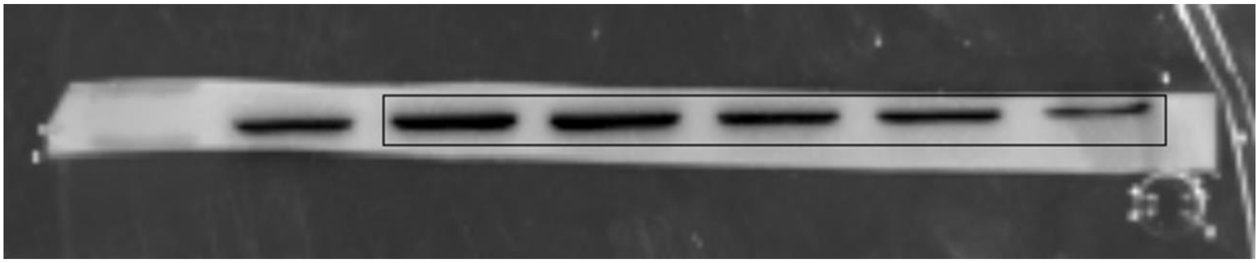

β-actin

Figure S6.Westernblot analyses of Bax, Bcl-2, LC3, P62 protein expression in samples with the full size panels. β-actin as controls were done and the protein is run consist with an accurate standard. Molecular weight of Bcl-2:26KDa, the lower band corresponds to Bcl-2.
